# Editorial: Cervical cancer: updates from the Mexican national consensus

**DOI:** 10.3389/fonc.2024.1493334

**Published:** 2024-09-27

**Authors:** Lucely Cetina-Pérez, Oscar Medina-Contreras

**Affiliations:** ^1^ Departamento de Investigación Clínica, Instituto Nacional de Cancerología, Mexico City, Mexico; ^2^ Modelo Integral para la Atención del Cáncer Cervicouterino Localmente Avanzado y Avanzado (MICAELA) Program, Instituto Nacional de Cancerología, Mexico City, Mexico; ^3^ Epidemiology, Endocrinology & Nutrition Research Unit, Mexico Children’s Hospital, Mexico City, Mexico

**Keywords:** HPV, cervical cancer, prevention, immunotherapy, GRADE system

Human papillomavirus (HPV) is a common virus that places a significant burden on low- and middle-income countries, particularly among women. Its high prevalence is a major public health concern and is straining healthcare systems worldwide. In response, the World Health Organization launched a global strategy in 2020 to accelerate the elimination of cervical cancer (CC) (1). Recognizing the severity of the problem, experts from the Mexican National Cancer Institute (NCI) assembled a multidisciplinary team to develop the National Consensus on Cervical Cancer (2023 update). These comprehensive consensus documents provide evidence-based recommendations for the medical community on the epidemiology, diagnosis, screening, treatment, immunotherapy, and prevention of CC (2–7), tailored to the specific context of Mexico. It serves as a valuable resource for practitioners caring for adult patients, excluding the pediatric population.

A modified Delphi process was employed, involving a general coordinator, 13 coordinators, and 62 experts in the relevant fields. An exhaustive search was conducted on PubMed using the search term “cervical cancer” combined with the keywords Epidemiology, Molecular Biology, Pathology, Prevention, Screening, Early Stage, Locally Advanced, Advanced Cancer, Immunotherapy, Histology, Probiotics, Mental Disease, Pain, and Radiotherapy. The search covered the period from January 2020 to April 2023. All publications were retrieved and reviewed by all consensus members. The quality of evidence and recommendations was assessed using the Grading of recommendations Assessment, Development, and Evaluation (GRADE) system (**Table 1**).

**Table 1:** The GRADE system.

Parameters	Code
Quality of evidence	
High	A
Moderate	B
Low	C
Very Low	D
Strenght of the recommendation	
Strongly for the intervention	1
Weakly forthe intervention	2
Weakly against the intervention	2
Strongly against the intervention	1

Coordinators developed 50 statements and voted anonymously on their content and quality using a Likert scale (totally agree, agree, uncertain, partially disagree, totally disagree). Statements with a totally agree score >75% were retained unchanged. Statements with a totally disagree score >75% were eliminated. Statements with a totally agree or totally disagree score <75% were revised. Consensus was achieved through multiple rounds of voting. The results of the most recent voting were presented on 27-28 September 2023 in a closed-doors session at the 41^st^ National Oncology Meeting SMEO, in Cancún, Mexico. In this session, statements with >75% approval were ratified, and statements with <75% approval were discussed to reach a consensus or to eliminate them. After all statements were voted on, all participants edited, reviewed, and approved their manuscripts.

Luvián-Morales et al. analyzed the risk factors for the development of CC, and identified coinfections with sexually transmitted conditions, genetics, cervicovaginal microbiota, nutrition, abnormal immune responses, smoking, and hormonal contraceptives, as non-independent cofactors for the development of CC. Vallejo-Ruiz et al. summarized the most recent molecular factors associated with CC. Interestingly, the authors highlighted a new link between the number of HPV DNA integrations and the cervical lesions. They also suggested new functions for the classical proteins E5, E6, and E7. Furthermore, the integration of genomic, transcriptomic, proteomic, metabolomic and epigenomic information provides novel molecular features along with potential biomarkers and therapeutic targets. González-Rodríguez et al. reviewed preventive measures, focusing on the effectiveness, long-term protection, and safety of HPV vaccination programs. They showed long-term protection and safety worldwide, using the available vaccines (bivalent, quadrivalent, and nonavalent) reaching a 54%-83% decrease in high-grade cervical abnormalities and genital warts. However, income disparities and vaccine availability are the main obstacles.

Barquet-Muñoz et al. analyzed the challenges faced by detection programs and screening tools in Mexico. The researchers highlighted the role of poorly organized screening programs in vulnerable groups that do not initiate or rarely undergo screening. They proposed cytology-based screening strategies to accurately identify women at higher risk for cervical cancer and to establish screening schedules. New detection tools, such as novel biomarkers or automated HPV detection in the vagina or urine, may improve screening coverage. Arango-Bravo et al. summarized the current scientific evidence to formulate clinical recommendations regarding the classification, diagnostic approach, and treatment of rare histologic subtypes of cervical cancer. The authors focused on a multimodal approach as critical for the treatment of tumors with special histologies.

Surgery is the first choice in the early stages of CC, as reviewed by Nájera-Muñoz et al. Despite being a radical procedure, hysterectomy is the standard treatment, but relies on the need to preserve fertility. The authors proposed a multidisciplinary evaluation to determine the therapeutic approach, aiming for less radical surgeries, and an individualized approach.

The intestinal microbiota has a crucial importance as one of the new factors participating in the regulation of the immune response. Gutiérrez-Salmeán et al. summarized the effects of supplementation with prebiotics, probiotics, and synbiotics on high-risk HPV infection, precancerous lesions, and CC development. They concluded that a higher dietary fiber intake leads to a reduced risk of HPV infection, while some probiotics, prebiotics, and synbiotics reduce adverse effects; therefore, they recommend conducting high-quality clinical trials to evaluate this effect.

Aguiar-Rosas et al. highlighted the importance of pain treatment in cases of cervical cancer, where pain is the most feared and disabling symptom. They provided relevant recommendations on pharmacology, interventional pain management, and the possible complications arising from the use of opioids in a comprehensive and individualized way, taking into account the timely and appropriate use of pharmacologic treatment in addition to interventional procedures.

While the consensus has led to extensive research on current practices, the investigation of innovative treatments such as individualized chemotherapy for patients with locally advanced cervical cancer, and the role of psycho-oncological support are essential and must not be overlooked (6). These studies, including one examining chemotherapy regimens in combination with radiotherapy, will be published at a later date.
